# Clinical, magnetic resonance imaging, and neuropathological features of suspected inborn methylmalonic aciduria in a domestic shorthair kitten

**DOI:** 10.1093/jvimsj/aalag150

**Published:** 2026-07-25

**Authors:** Chloe Fisher, Sara Degl’Innocenti, Ana Ortiz, Alison Major, Faye Rawson, Giulia Scarin, Angela Fadda

**Affiliations:** Eastcott Veterinary Referrals, Linnaeus Group, Swindon SN3 3FR, United Kingdom; Veterinary Pathology Service, University of Nottingham, Nottingham LE12 5RD, United Kingdom; San Raffaele-Telethon Institute for Gene Therapy, GLP Test Facility, IRCCS San Raffaele Scientific Institute, Milan 20129, Italy; Department of Veterinary Medicine, University of Cambridge, CB3 0ES, United Kingdom; Langford Vets—Bristol Veterinary School, University of Bristol, Langford, Bristol BS40 5DU, United Kingdom; Langford Vets—Bristol Veterinary School, University of Bristol, Langford, Bristol BS40 5DU, United Kingdom; Veterinary Pathology Service, University of Nottingham, Nottingham LE12 5RD, United Kingdom; Langford Vets—Bristol Veterinary School, University of Bristol, Langford, Bristol BS40 5DU, United Kingdom

**Keywords:** feline, metabolic brain disease, neuroanatomy, neuropathology

## Abstract

A 10-month-old domestic shorthair kitten was evaluated for progressive neurological dysfunction characterized by ataxia, episodic opisthotonus, impaired vision, dysphagia and seizures. Clinicopathological findings included marked hypocobalaminemia and abnormal concentrations of urinary methylmalonic acid, methylcitrate, and propionylglycine. Magnetic resonance imaging revealed generalized cerebral and cerebellar atrophy, with mild ventriculomegaly. Despite dietary modification, cobalamin supplementation, antiepileptic therapy, neurological deterioration progressed, culminating in refractory seizures and euthanasia. Post-mortem examination revealed multiorgan and central nervous system changes, characterized by neuronal degeneration and necrosis, cortical atrophy, globular eosinophilic intracytoplasmic material within neurons and gliosis. Methylmalonic aciduria is a rare metabolic disorder resulting from defects in cobalamin metabolism or methylmalonyl-CoA mutase activity.

## Case history

A 10-month-old, entire male, domestic shorthair kitten was referred for a 7-day history of reluctance to jump, inappropriate toileting, and difficulty eating despite a good appetite. Acute deterioration, with episodes of opisthotonus triggered by handling, developed within 24-h of referral. The kitten was smaller and less active than its littermate raised under identical conditions. The cat was fed a complete commercial diet (supermarket own brand, kitten, chicken and rice, dry) and had supervised outdoor access. Initial investigations by the referring veterinarian included thoracic and abdominal radiographs that revealed normal bone density, and laboratory testing. On examination, bodyweight was 1.99 kg (body condition score 3/9). Neurological assessment revealed ambulatory tetra-ataxia, wide-based stance, impaired menace responses, reduced visual placing and tracking with normal pupillary light reflexes, spontaneous vertical nystagmus and episodic opisthotonus on elevation of the head. Neuroanatomical localization was consistent with multifocal intracranial disease involving the forebrain, cerebellum, and bilateral vestibular system.

Initial laboratory testing identified mild macrocytic normochromic anemia (5.49; RI 6-10.1), mean cell volume (51.1 fL; RI 41.3-52.6), lymphopenia (1.81; RI 2-7.2), low creatinine (74 umol/L; RI 133-175) and total protein (54.3 g/L; RI 75-88), alanine aminotransferase (57 U/I; RI 15-45), phosphate (2.13 mmol/L; RI 0.95-1.55) and glucose (8.2 mmol/L; RI 3.5-5) above the reference range, and plasma ammonia above the reference interval (51.9 umol/L; RI 0-50). Blood smear examination revealed anisocytosis, macrocytosis, and hypersegmented neutrophils. Serum cobalamin concentration was markedly decreased (63 pmol/L; RI 231-617), folate was increased, and trypsin-like immunoreactivity was mildly reduced (9.6 ng/mL; 12.1-82). Venous blood gas analysis showed no metabolic acidosis.

Magnetic resonance imaging of the brain revealed generalized cerebral atrophy with widened sulci, rounded gyri, atrophy of the caudal third of the cerebellum and mild symmetrical ventriculomegaly (Figure 1). Abnormalities were not detected on cerebrospinal fluid analysis (clear colorless fluid, no nucleated cells, total protein 20 mg/dL). Infectious disease testing, including feline coronavirus and *Toxoplasma gondii* serology and cerebrospinal fluid polymerase chain reaction, was negative.

**Figure 1:**
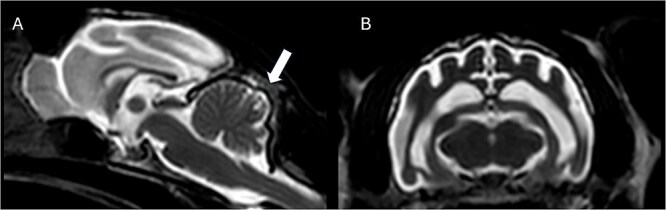
(A) Sagittal T2-weighted. (B) Transverse T2-weighted magnetic resonance images of the cat’s brain showing generalized cerebral atrophy with widened sulci, rounded gyri, atrophy of the caudal third of the cerebellum (arrow) and symmetrical ventriculomegaly.

Urinary organic acid analysis demonstrated methylmalonic acid concentration above the reference interval (>2000 μmol/mmol creatinine; RI 0-20) with methylcitrate and propionylglycine also above their reference intervals; malonic acid was within laboratory reference limits.

The kitten was initially managed with a low-protein wet diet, parenteral hydroxocobalamin (0.2 mg/kg subcutaneously, once daily for 5 days) and thiamine (10 mg subcutaneously, once daily, for 5 days). This was followed by oral cobalamin supplementation (1/2 Cobaloplex capsule once daily) and L-carnitine (250 mg once daily) to continue at home following discharge.

The cat showed progressive neurological deterioration in mentation and markedly reduced play behaviors. Repeated examination after 10 days identified exacerbation of the previously described tetra-ataxia and spontaneous nystagmus, increased frequency of the opisthonic episodes with the addition of fine, vertical head tremor. The cat developed recurrent myoclonic and generalized tonic–clonic seizures. Levetiracetam (60 mg/IV loading dose followed by maintenance doses of 20 mg/kg every 8 h and phenobarbitone 2.5 mg/kg every 12 h) were administered for seizure control.

An electroencephalogram showed large periods of abundant, generalized rhythmic slowing within the delta frequency band (1-2 Hz) of high amplitude (>150 μv), in addition to high amplitude spike and spike and slow wave complexes. During the examination, the cat experienced a seizure.

Myoclonic seizures reoccurred in cluster and progressed to generalized tonic–clonic seizure activity. Generalized tonic–clonic seizures failed to respond to anticonvulsant treatment and additional phenobarbitone IV (4 mg/kg) doses and were controlled with induction of general anesthesia (propofol constant rate infusion 0.4 mg/kg/h). Due to the poor prognosis and progressive neurological decline, further testing was not authorized by the owner and euthanasia was elected.

The brain, spinal cord, heart, and sections of liver and muscle were removed for post-mortem histopathological examination. The heart, liver, skeletal muscle (gluteal and quadriceps), and central nervous system (cerebellum, cerebrum, and spinal cord) were examined. Grossly, moderate brain atrophy accompanied by mild dilation of the lateral ventricles, mesencephalic aqueduct and fourth ventricle was observed. Microscopically, the cerebellar cortex was diffusely atrophied, with slight thinning and pallor of the cerebellar folia due to decreased cellularity, most prominent in the granule cell layer (Figure 2). The neuroparenchyma was mildly hypercellular, with increased numbers of glial cells. Scattered throughout all the sections, there was multifocal, mild vacuolation of the white matter (spongiosis), some neurons appeared shrunken and fragmented with loss of Nissl substance, and a hyper-eosinophilic cytoplasm with a pyknotic nucleus, consistent with neuronal necrosis (Figure 3). Within the cytoplasm of multiple neurons, within cerebral and cerebellar cortex, a granular to globular, bright eosinophilic to red material was observed (Figures 4 and 5). Purkinje cells were irregularly spaced with scattered necrosis and loss, and often contained abundant, granular to globular, eosinophilic to red intracytoplasmic material, occasionally displacing the nucleus peripherally and markedly distending the perikarya. This material did not stain with Periodic acid-Schiff stain (PAS) and stained inconsistently with Luxol fast blue stain. Histopathological changes in other examined organs included mild myocardial fibrosis, hepatocellular degeneration, skeletal muscle atrophy, and degeneration.

**Figure 2:**
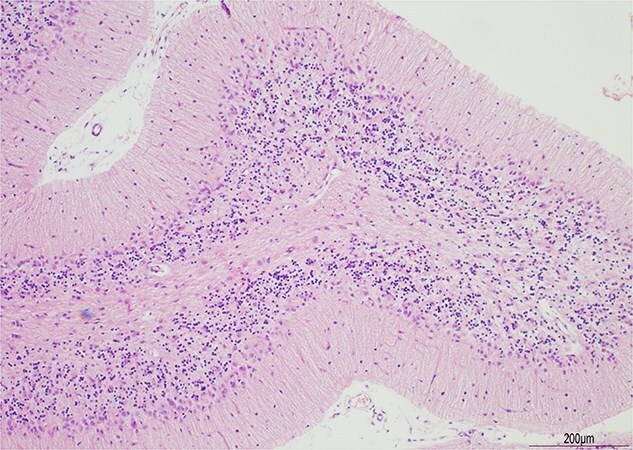
Cerebellar cortex. Note the thinning of the cerebellar folia with decreased cellularity prominently affecting the granule cell layer. Hematoxylin and eosin. Bar 200 μm.

**Figure 3:**
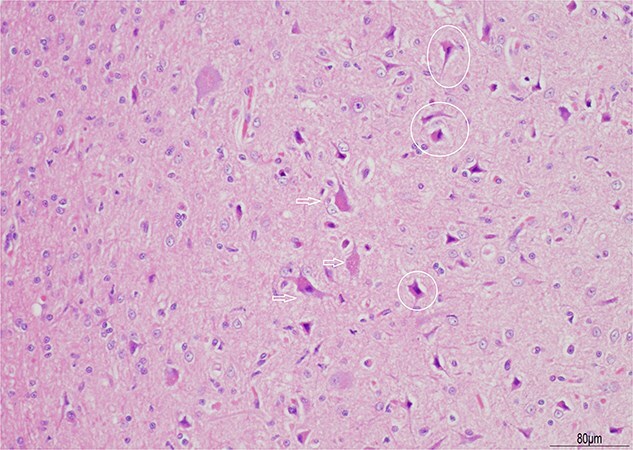
Cerebral cortex. Note the multifocal necrotic neurons (white circles), some of which containing granular, deeply eosinophilic to red, intracytoplasmic material (white arrows). Hematoxylin and eosin. Bar 80 μm.

**Figure 4:**
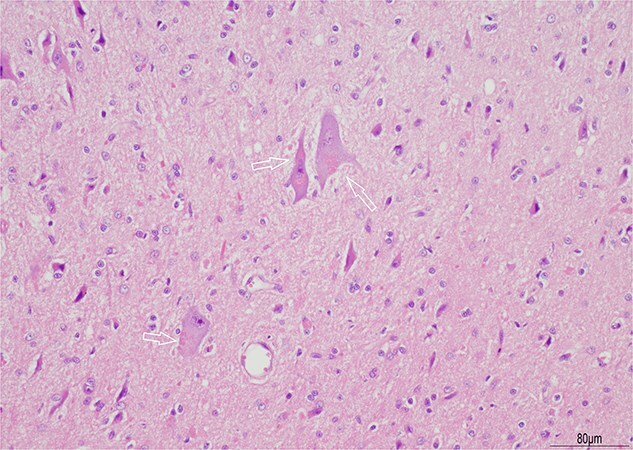
Cerebral neurons containing variable amounts of intracytoplasmic, granular to globular, deeply eosinophilic to red material (white arrows). Hematoxylin and eosin. Bar 80 μm.

**Figure 5:**
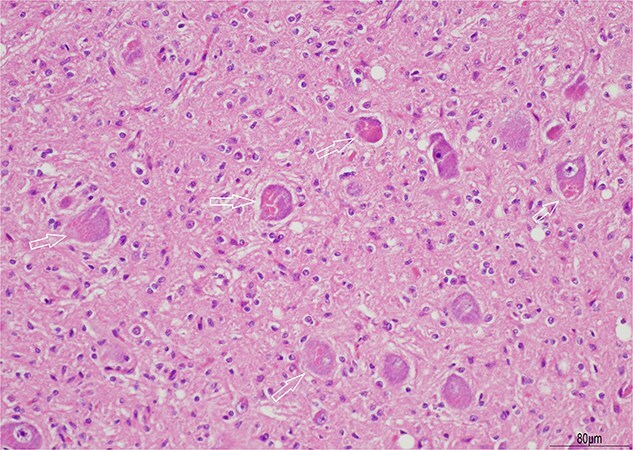
Cerebellar nuclei neurons containing variable amounts of intracytoplasmic, granular to globular, deeply eosinophilic to red material (white arrows). Hematoxylin and eosin. Bar 80 μm.

## Discussion

This report presents a detailed characterization of the neurological presentation and magnetic resonance imaging (MRI) abnormalities, and documents the neuropathological findings in cat with suspected inborn methylmalonic aciduria and hypocobalaminemia. Methylmalonic acidaemia/aciduria (MMA) can result in severe neurological signs in people and animals^1–4^ and is associated with nutritional, acquired, and inborn deficiencies of cobalamin (CBL). Deficiencies resulting in a lack of intracellular CBL cause an intracellular and systemic accumulation of methylmalonic acids, resulting in MMA. Concurrently, hypocobalaminemia can interfere with hematopoiesis, resulting in anemia and pancytopenia and inhibit carbamoyl phosphate synthetase I activity, an enzyme of the urea cycle that metabolizes ammonia to carbamoyl phosphate resulting in hyperammonemia. The elevation of both methylmalonic acids and ammonia plasma concentrations likely underlie neurological signs, though the exact mechanisms remain unclear.^5^

In humans, MMAs comprise a genetically heterogeneous group of disorders of methylmalonate and cobalamin metabolism. They might arise from inborn errors of cobalamin absorption, intracellular trafficking, defects in remethylating, or impaired activity of methylmalonyl-CoA mutase. More than 80 pathogenic mutations have been described for defects in methylmalonyl-CoA mutase alone.^1^^,^^6^

In several breeds of dogs, congenital hypocobalaminemia associated with MMA and variable hyperhomocysteinemia have been described with mutations in CUBN and AMN genes, comparable to human Imerslund–Gräsbeck syndrome.^7^^,^^8^ In addition, a predisposition to develop cobalamin deficiency has been established in many other canine breeds.^9^

In cats, hypocobalaminemia associated with MMA is mainly acquired, secondary to exocrine pancreatic insufficiency, intestinal diseases such as chronic inflammatory enteropathy and lymphoma, biliary disease, or combinations of these disorders.^10^^,^^11^ Congenital disorders of cobalamin absorption appear to be rare in this species, with only 2 suspected inborn cases published.^2^^,^^3^

Clinical signs related to cobalamin deficiency in cats are most often secondary to underlying diseases and include anorexia, vomiting, diarrhea, and impaired growth, although neuropathological sequalae associated with severe myelopathy have been previously reported in a cat affected by CBL and Folate deficiency secondary to pancreatitis.^12^ Neurological manifestations, reported only anecdotally and in association with MMA, are the predominant clinical feature in presumed congenital cases.^2^^,^^3^ Clinical improvement is generally expected for the acquired forms in people,^6^ dogs^13^ and cats^14–16^ upon resolution of the primary underlying conditions and CBL supplementation. However, death was reported for the 2 cats^2^^,^^3^ affected by neurological manifestation secondary to suspected inborn hypocobalaminemia and MMA.

Similarly to these 2 previously reported cases of suspected inborn MMA, the cat described in the present report experienced progressive deterioration, with worsening neurological signs ultimately leading to euthanasia. Dietary deficiency of CBL was unlikely in the cat of this current report, which was fed with a complete commercial diet. Although laboratory findings of high folate and low Serum trypsin‑like immunoreactivity (TLI) could not entirely exclude an early or mild stage of exocrine pancreatic insufficiency (EPI), empiric treatment and CBL supplementation did not lead to improvement of the clinical signs. Due to the rapid deterioration in clinical signs, which ultimately led to the decision for euthanasia, follow-up organic acid analysis or further testing was not performed. This represents a limitation, as in cases of cobalamin malabsorption urinary methylmalonic acid would be expected to decrease markedly after supplementation, providing diagnostic clarification. While it has been suggested that cats failing to respond adequately to enzyme and CBL supplementation might have concurrent undetected small intestinal disease,^17^ no macroscopic abnormalities of the intestine or pancreas were observed at necropsy. However, histopathological examination of these organs was not performed.

The most frequent hematobiochemical alterations observed under CBL deficiency in cats are non-regenerative anemia, leukopenia, hypoglycemia, and hyperammonemia,^17^ the latter being considered linked to the neurological manifestation.^15^^,^^16^ Conversely, neither hypoglycemia nor severe hyperammonemia was detected in the cat of this report. Interestingly, are also reported in a dog^4^ and a cat^2^ with neurological signs associated with suspected inborn MMA and hypocobalaminemia.

Given the young age of the cat, the refractoriness to treatment, the severity of neurological signs, the laboratory and radiological findings, an inborn MMA was suspected as the underlying cause of the clinical signs in the cat described in this report.

In people, inborn MMA are associated with severe metabolic and neurological signs. Despite advances in the treatment of MMA, mortality and disability rates remain high,^5^ particularly because of long-term sequelae. Main clinical manifestations include feeding difficulties, intellectual disability, psychomotor retardation, ataxia, abnormal muscle tone, pyramidal signs, spasticity, seizures, epilepsy, and lethargy.^5^^,^^6^^,^^18^

The presentation varies with age of onset: fulminant forms with metabolic crises and seizures occur predominantly in pediatric patients,^1^ whereas chronic forms led to disabilities resulting from defective nervous system development,^5^ and multiorgan failures with cardiac, renal manifestations, diabetes, and recurrent pancreatitis.^18^

The clinical signs observed in this cat included dysphagia, opisthotonus, ataxia, reduced vision, lethargy, and seizures, resembling those reported in MMA in humans.^19^ Interestingly, extensor rigidity, neck extension and dysphagia in the suspected case of inborn MMA in a dog.^4^

Available guidelines in humans recommend to run essential biochemical parameters in the acute setting to discriminate between a metabolically stable or a decompensating situation.^18^ Characteristic laboratory abnormalities during metabolic crises, such as metabolic acidosis, ketonemia/ketonuria, lactic acidosis, hypoglycemia, and hyperammonemia, might be absent in the chronic phases of the disorder.^1^ Since laboratory testing was not repeated at the cat’s last hospitalization, a raise in plasma ammonia coinciding with neurological deterioration cannot be excluded.

In this cat, main biochemical findings included abnormal MMA, alongside abnormal concentrations of methylcitrate and propionylglycine. Such profile is consistent with the diagnosis of inborn MMA in people, although propionylglycine levels are not pathognomonic.^20^ Growing evidence suggests that organic acids accumulating in MMA act synergistically to cause neurological damage through mechanisms such as impaired energy metabolism, oxidative stress, neuroinflammation, and excitotoxicity. Although MMA itself is not considered the primary toxic compound, it contributes to the inhibition of the respiratory chain and the tricarboxylic acid cycle via propionyl-CoA and its alternative metabolites.^20^ Specifically, methyl citrate is now recognized to have a major role in the insult to the nervous tissue.^20^

In people, final diagnosis of MMA relies on molecular testing to identify associated mutations.^21^ Functional assays on fibroblasts or lymphocyte can also be used to assess enzyme activity and cobalamin responsiveness.^18^ In this cat, the suspicion of a neurodegenerative inborn condition was further supported by radiological and neuropathological findings.

MRI of the cat’s brain showed generalized cerebral atrophy with marked widening of the sulci, rounding of the gyri, atrophy of the caudal third of the cerebellum, and symmetrical ventriculomegaly but lacked the symmetrical bilateral T2-weighted hyperintensities typically associated with metabolic disorders previously described in this species.^22^ In human MMA patients,^23^ cerebral atrophy and ventricular dilation, which represent the most important imaging abnormalities, are often accompanied by periventricular white matter changes while symmetrical bilateral caudate nuclei involvement has been described only in a minority of cases.^24^

By contrast, the only MRI description of a suspected acquired feline hypocobalaminemia and hyperammonemia^16^ consisted of diffuse, bilaterally symmetrical, T2-weighted hyperintense lesions, predominantly affecting the gray matter, in the thalamus, mesencephalon, pons, caudal cerebellar peduncles, and interposital nuclei. In that case, the cat had normal methylmalonic acid levels which was attributed to inappropriate sample storage. Nevertheless, the differences observed might reflect distinct pathogenetic and neurotoxicological mechanisms between acquired and inborn cobalamin deficiencies in cats.

Histopathological examination in this cat revealed multiorgan changes, partly resembling long-term abnormalities reported in human patients with MMA as a consequence of mitochondrial dysfunction. These included mild, myocardial interstitial fibrosis, liver alterations, and widespread brain pathology consistent with neuronal degeneration and necrosis, cortical atrophy, gliosis and mild multifocal white matter vacuolation.^5^^,^^25^ Similar findings have been described in a dog with suspected inborn MMA and malonic aciduria, including mild skeletal muscle denervation and brain atrophy with ventricular dilation.^4^White matter vacuolation, however, appears to be as well predominant finding in a cat with acquired CBL deficiency^12^ and in an experimental feline model of subacute combined degeneration, a condition associated with disturbances of cobalamin metabolism in people.^26^

In children older than 10 months, additional neuropathological features of MMA have been reported, such as reactive gliosis, hypomyelination, multifocal cerebellar hemorrhages, and depletion or underdevelopment of the cerebellar external granule cell layer.^24^ In the cat reported here, neuronal necrosis was accompanied by granular to globular eosinophilic intracytoplasmic material within neurons and Purkinje cells, raising congenital storage diseases as differential diagnoses.

Although the neuropathogenesis of MMA remains unclear, MMA and related metabolites induce cell damage in various neuronal culture systems. Therefore, their accumulation in the brain is considered the major pathogenic factor. Such accumulation leads to mitochondrial dysfunction, including energy failure and oxidative stress, as well as neuroinflammatory responses and excitotoxicity, ultimately activating cell death pathways.^20^ MMA also inhibits lactate dehydrogenase, thereby disrupting the astrocyte-neuron lactate shuttle and compromising neuronal energy metabolism.^27^ Together, these mechanisms contribute to progressive neural damage.^20^

Despite exclusion of neuronal ceroid lipofuscinosis, full characterization of the intracytoplasmic material was not achieved, however, other lysosomal storage diseases were considered less likely in view of the abnormal clinicopathological and biochemical findings. Additional investigations such as electron microscopy, analysis of lectin binding patterns, histochemical assays, and special stains (eg, cytochrome c oxidase and succinate dehydrogenase for mitochondrial disease) could have helped to identify more specifically the nature of the defect, similarly, gliosis and Purkinje cell loss were assessed only on H&E sections, without immunohistochemistry (IHC)-based or quantitative confirmation.

This report is subject to limitations, primarily related to the incomplete diagnostic investigations. Although an inborn methylmalonic aciduria is strongly suspected, confirmation would have required genetic or functional enzyme testing. Antemortem diagnosis could also have been strengthened by repeating laboratory investigations after parenteral cobalamin supplementation. In addition, the lack of pancreatic and gastrointestinal histopathology does not fully exclude acquired form.

## Conclusion

Despite the limitations outlined above, these findings strongly support the diagnosis of an inborn error of metabolism rather than acquired hypocobalaminemia in this cat and highlight the diagnostic and therapeutic challenges posed by signs of neurological disease associated with MMA and hypocobalaminemia in cats.

## Off-label antimicrobial declaration

Authors declare no off-label use of antimicrobials.

## Institutional animal care and use committee or other approval declaration

This case report involved a client-owned animal and was conducted in accordance with best practice veterinary care. Informed owner consent was obtained for diagnostic investigations and post-mortem examination.

## Abbreviations

BCS: body condition score
CBL: cobalamin
COX: cytochrome c oxidase
CRI: constant rate infusion
MMA: methylmalonic aciduria
MRI: magnetic resonance imaging
SDH: succinate dehydrogenase
